# Three-dimensional whole-liver perfusion magnetic resonance imaging in patients with hepatocellular carcinomas and colorectal hepatic metastases

**DOI:** 10.1186/1471-230X-13-53

**Published:** 2013-03-25

**Authors:** Sheng-Xiang Rao, Cai-Zhong Chen, Hao Liu, Meng-Su Zeng, Xu-Dong Qu

**Affiliations:** 1 Department of Diagnostic Radiology, Zhongshan Hospital, Fudan University, and Shanghai Medical Imaging Institute, Shanghai, People’s Republic of China; 2 Department of Interventional Radiology, Zhongshan Hospital, Fudan University, and Shanghai Medical Imaging Institute, Shanghai, People’s Republic of China

**Keywords:** 3D Magnetic Resonance Imaging, Perfusion Imaging, HCC, Colorectal hepatic metastasis, Inter-observer analysis

## Abstract

**Background:**

Three-dimensional (3D) whole-liver perfusion magnetic resonance(MR) imaging with parallel imaging, a novel imaging method to characterize tumor vascularization *in vivo,* has recently been applied to comprehensively image perfusion changes in large tumors. Coupled with new perfusion software, this technique enables motion correction, registration, and evaluation of perfusion MR parameters. The purpose of this study was to assess the feasibility of 3D whole-liver perfusion MR, for imaging hepatocellular carcinoma (HCC) and colorectal hepatic metastases (CRHM).

**Methods:**

26 patients with hepatic tumors (10 HCC; 16 CRHM) were subjected to 3D whole-liver perfusion MR with a temporal resolution of 3.7 seconds. The following estimated perfusion parameters were measured: the volume transfer constant K^trans^ (min^-1^); the volume (V_e_) of extravascular extracellular space (EES) per volume unit of tissue; and the flux rate constant between EES and plasma K_ep_ (min^-1^). Statistical analysis was conducted to investigate inter-observer characteristics and significance of the measured parameters.

**Results:**

Inter-observer agreement analysis (95% limits of agreement) yielded a mean difference of −0.0048 min^-1^ (−0.0598 ~ 0.0502) for K^trans^ , -0.0630 ml (−0.5405 ~ 0.4145) for V_e_, and −0.0031 min^-1^ (−0.0771 ~ 0.0709) for K_ep_ respectively. When comparing images from patients with HCC vs. CRHM, significant differences were seen for the mean K^trans^ (p = 0.017), but not for V_e_(p = 0.117) or K_ep_(p = 0.595).

**Conclusion:**

Herein we show that 3D whole-liver MR perfusion imaging with semi-automatic data analysis is feasible and enables the reliable quantitative evaluation of the perfusion parameters for HCCs and CRHMs.

## Background

The progression of novel therapeutic strategies for advanced liver cancer stimulates continued development of diagnostic tools. Whereas existing imaging techniques are largely limited to morphological evaluation, perfusion imaging has the potential to provide more comprehensive tumor characterization [1]. Several perfusion imaging modalities have been tested to image liver tumors, including contrast-enhanced ultrasound [2], computed tomography (CT) [3,4] and magnetic resonance (MR) imaging [5-7]. Although several of these modalities use contrast injection, which requires the patient to have adequate renal function, perfusion MR imaging is noninvasive [5-9]. To increase applicability and reproducibility of this technique, Tofts et al. [9] standardized the estimation of kinetic parameters derived from perfusion MR data. These include a) the volume transfer constant (K^trans^ [min^-1^]); b) the volume (V_e_) of extravascular extracellular space (EES) per volume unit of tissue; and c) the flux rate constant between EES and plasma (K_ep_ [min^-1^]). The rate constant is the ratio of the transfer constant to the EES (K_ep_ = K^trans^/V_e_). Currently, the majority of perfusion MR studies focus on a specific area of tumor, failing to provide comprehensive evaluation of the tumor as a whole. However, the recently developed technique of 3D whole-liver perfusion MR imaging, combined with parallel imaging technique, supports imaging of the entire liver, depicting overall changes in liver perfusion as well as tumor-specific flow, which also enables visualization of hemodynamic interactions between the tumor and the surrounding liver tissue [5,7]. In addition, new perfusion software enables registration and correction of motion, reducing misregistration and respiratory artifacts, and permitting evaluation of parameters such as K^trans^, V_e_, and K_ep_.

Herein, we evaluate the inter-observer variation during semi-automated measurement for three-dimensional (3D) whole-liver MR perfusion parameters. In addition, this study specifically addresses the significance of observed differences between perfusion imaging of HCC and CRHM using this novel technique.

## Methods

### Patient and tumor characteristics

Twenty-six patients with solid liver lesions were included in this study, consisting of 10 cases of HCC (7 male, 3 female, aged 45–75 years old [mean age 58.8 years]) and 16 cases of CRHM (11 male, 5 female, aged 48–67 years old [mean age,61.3 years]). In HCC patients, the diagnosis was either histologically confirmed during surgery (n = 8) or based on dynamic CT findings together with high serum α-fetoprotein (n = 2). Cirrhosis was confirmed in all patients with HCC (Child-Pugh A: 9, B: 1), either through pathological assessment or clinical and imaging criteria. Of the 16 CRHM patients, four cases were histologically confirmed during surgery. Twelve cases showed significant growth or regression of lesions at follow-up imaging after the commencement of chemotherapy (defined as ≥20% diametric growth or regression, mean time of follow-up 4.2 months with a range of 2–6 months). All patients were required to not have received any therapy prior to inclusion. In each patient, a single lesion was chosen as study target, which were required to be well demarcated contrast-enhancing solid masses larger than 1.5 cm in the longest diameter. This study was approved by the ethics committee of Zhongshan Hospital, Fudan University (Approval No:2010-17) in compliance with the Helsinki Declaration, and written informed consent was obtained from the patient for publication of this report and any accompanying images.

### Perfusion MR imaging

**Figure 1:**
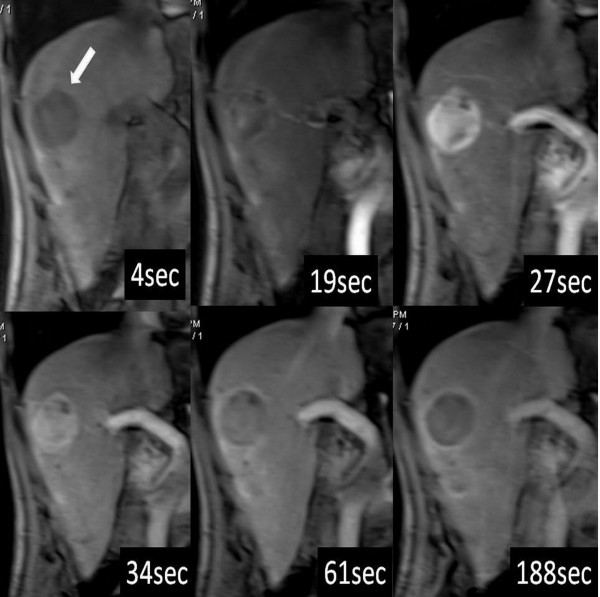
Perfusion MR images of the liver in a 49-year-old male patient with HCC, which showed classic enhancement features of hyper-vascularity at arterial phase and washout at portal venous phase and delayed phase by selected time points from 32 measurements.

The patients were asked to fast for 6 hours before imaging. Perfusion MR was performed using a 3.0-T system with phased-array coils (Verio, Siemens Medical Solutions, Erlangen, Germany). For T_1_ map calculation, a dual flip angle (FA) 3D gradient echo sequence with volumetric interpolated breath-hold examination.

(VIBE) was performed preceding injection of contrast material. The following imaging parameters were used: 2.84/1.02 (repetition time msec/echo time msec), 2° and 9° FA, 134 × 256 matrix, 2.5 × 1.5-mm in-plane pixel size, 380 × 333-mm field of view, 20 cm slab thickness resulting in an interpolated 5-mm section thickness, and 750 Hz/pixel bandwidth. A parallel imaging technique (R factor of two) was performed using generalized autocalibrating partially parallel acquisition. Whole-liver perfusion MR imaging was performed by VIBE in the coronal plane using 9° FA. The geometrical and spatial parameters of the perfusion series match the pre-contrast sequence (resolution, dimensions, orientation) with a temporal resolution of 3.7 seconds. The first “breath-hold” lasted 1 scan, which was aligned with the beginning of contrast material injection to obtain a baseline scan. At the 16th second, three subsequent “breath-holds” were orchestrated, each lasting for 5 scans every 8 seconds. Finally, 4 subsequent “breath-holds”, each accounting for 4 scans were obtained in 12-second intervals, resulting in the acquisition of a total of 32 scans (Figure 1). All patients received 0.1 mmol/kg body weight of gadopentetate dimeglumine, administered intravenously at 5 mL/s through a cubital or cephalic venous entry, and flushed with 20 mL 0.9% saline.

### Data analysis

**Figure 2:**
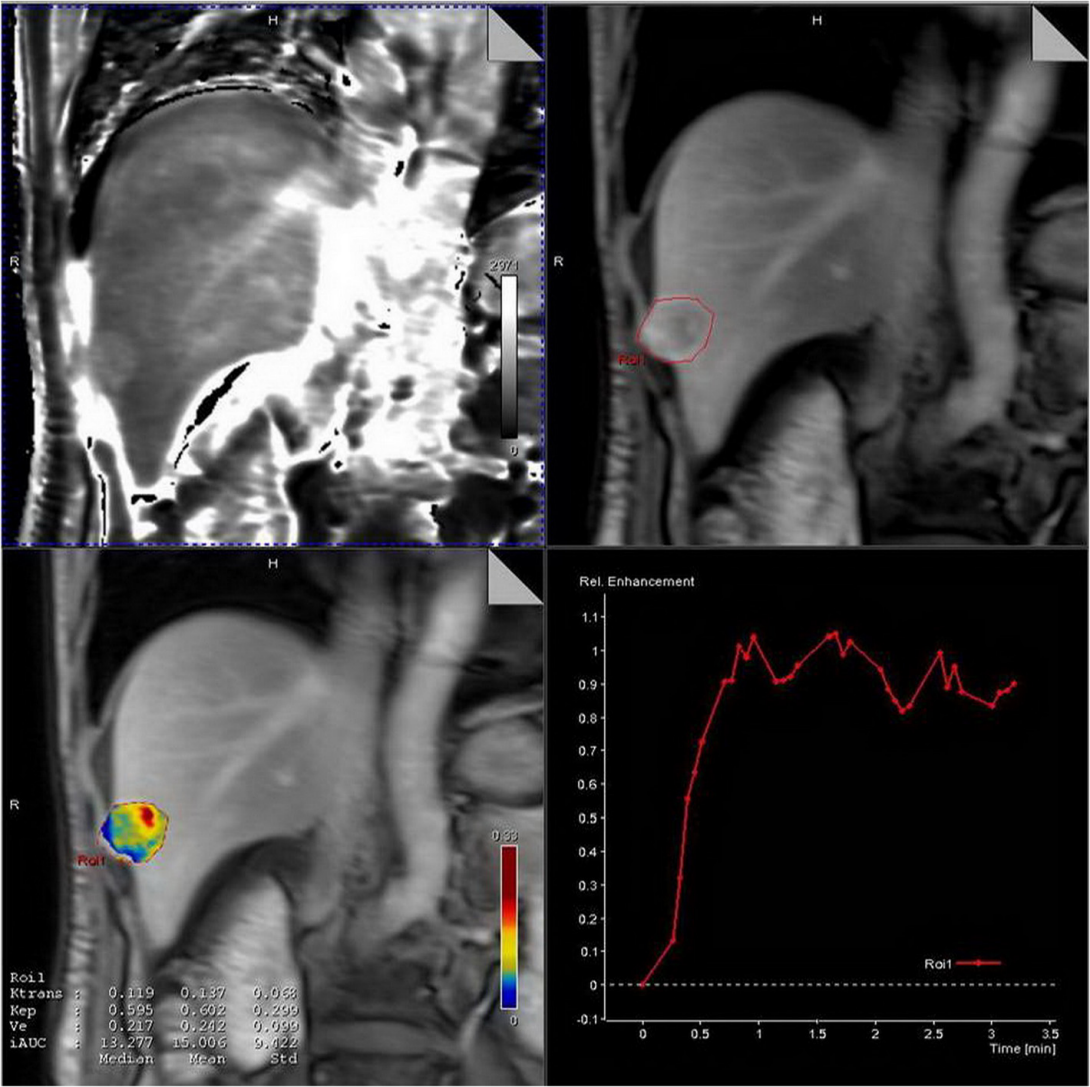
**Example of evaluation for perfusion parameter by the Tissue 4D tool.** T1 map is automatically calculated by the precontrast images (upper left),and region of interest(ROI) is drawed covering the tumor on the dynamic contrast phases (upper right), then the time-intentsity curve is automatically calculated(lower right).Finally the colored-code parametric map of MR perfusion is generated(lower left).

MR images were analyzed by using commercially available software on a separate workstation (Tissue 4D, Syngo MR B17, Siemens Healthcare) to calculate the perfusion parameters. Semi-automatic evaluation of hepatic tumors was performed by two radiologists experienced in abdominal MR imaging, who separately and independently evaluated the hepatic tumors. The following algorithm was applied: 1. Motion correction: selection of the the first volume of the dynamic series as reference volume. The new motion-corrected series automatically replaces the original dynamic series; 2. Registration: selection of the first volume in the dynamic series as reference volume for registration; 3. Curve calculation: calculation of enhancement curves and segment structures manually by drawing regions of interest (ROIs) in the dynamic series. The curves are shown as relative enhancement curves with the first volume serving as reference. 4. Pre-evaluation: the T_1_ map calculation of pre-contrast data is a prerequisite for pharmacokinetic modeling. It runs automatically when we start the pre-evaluation blind. T_1_ fitting is restricted to pixels with values above the noise level cutoff. Once the T_1_ map is calculated, the pre-contrast is replaced; 5. Evaluation: the Evaluation blind allows pharmacokinetic modeling of the mean curves for the selected ROI label. The two-compartment Tofts model of arterial input functions (AIFs) is provided; 6. Results: the ROI functionalities on the Results blind comply with the functionalities on the Curve Calculation blind. The setting affects only the ROIs displayed in parameter images (Figure 2); 7. Export: export of the results.

### Statistical analysis

Inter-observer variation for perfusion parameters (K^trans^, V_e_, and K_ep_) was analyzed using a Bland-Altman plot. Mann–Whitney test and independent samples t-test were conducted to analyze the difference in mean parameter values between HCC vs. CRHM patients. All statistical analyses were conducted using MedCalc (MedCalc for Windows, version 9.6.4.0). A p-value of less than 0.05 was considered significant.

## Results

### Morphological features

The mean overall tumor size was 42.6 mm (range, 15–96 mm), with a mean of 55.4 mm (range, 31.3-96 mm) for HCC and 42.7 mm (range, 15–95 mm) for CRHM. On the arterial phase HCC tumors were either homogenously (n = 3, 30%) or heterogeneously (n = 7, 70%) hyperintense; while CRHM were peripherally (n = 7, 43.8%) or heterogeneously (n = 9, 56.2%) hyperintense. On portal phase and delayed phase, HCCs were hypointense (n = 9, 90%) or isointense (n = 1, 10%); while the CRHM were peripherally (n = 7, 43.8%) or heterogeneously (n = 9, 56.2%) hyperintense. The enhancement pattern during the dynamic study revealed that the HCCs showed arterial enhancement with washout (n = 9, 90%) or without (n = 1, 10%) during the portal or delayed phase. In contrast, CRHM displayed either progressive heterogeneous enhancement (n = 9, 56.2%) or progressive peripheral, rim-like enhancement (n = 7, 43.8%).

### Quantitative findings during perfusion MR

**Figure 3:**
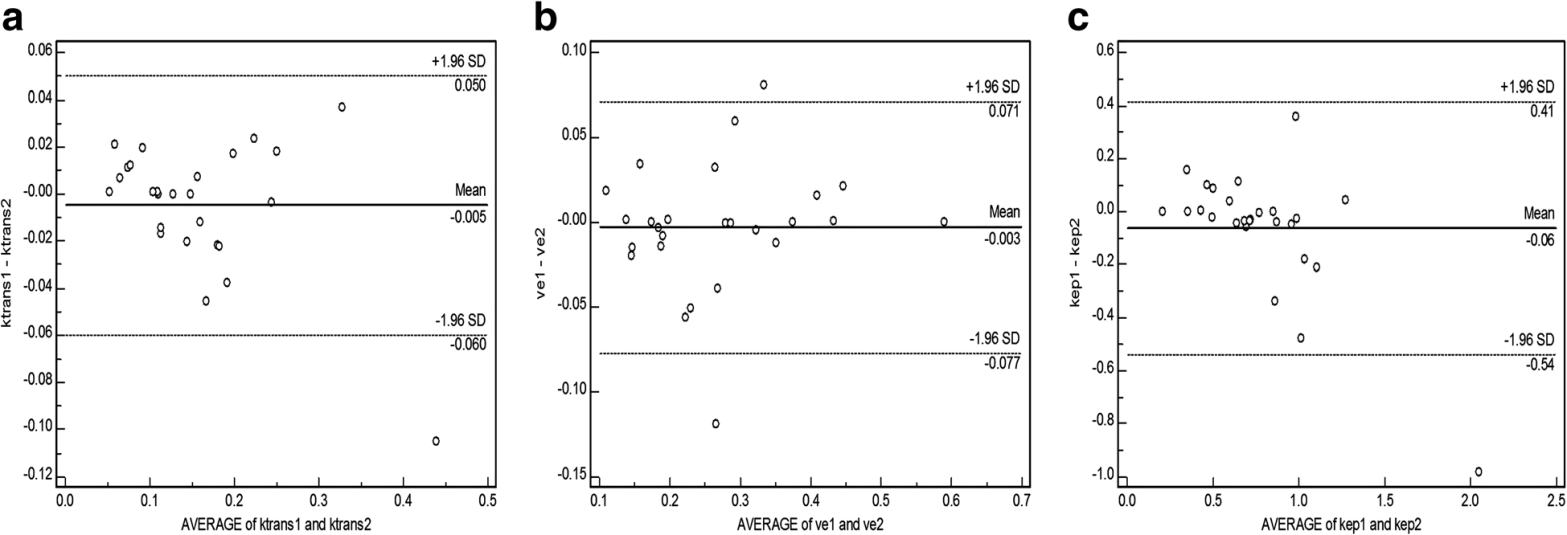
**a-c. Bland-Altman plots showing the difference for Ktrans (Figure**3**a), Ve (Figure**3**b), Kep (Figure**3**c) between the two readers.**

**Figure 4:**
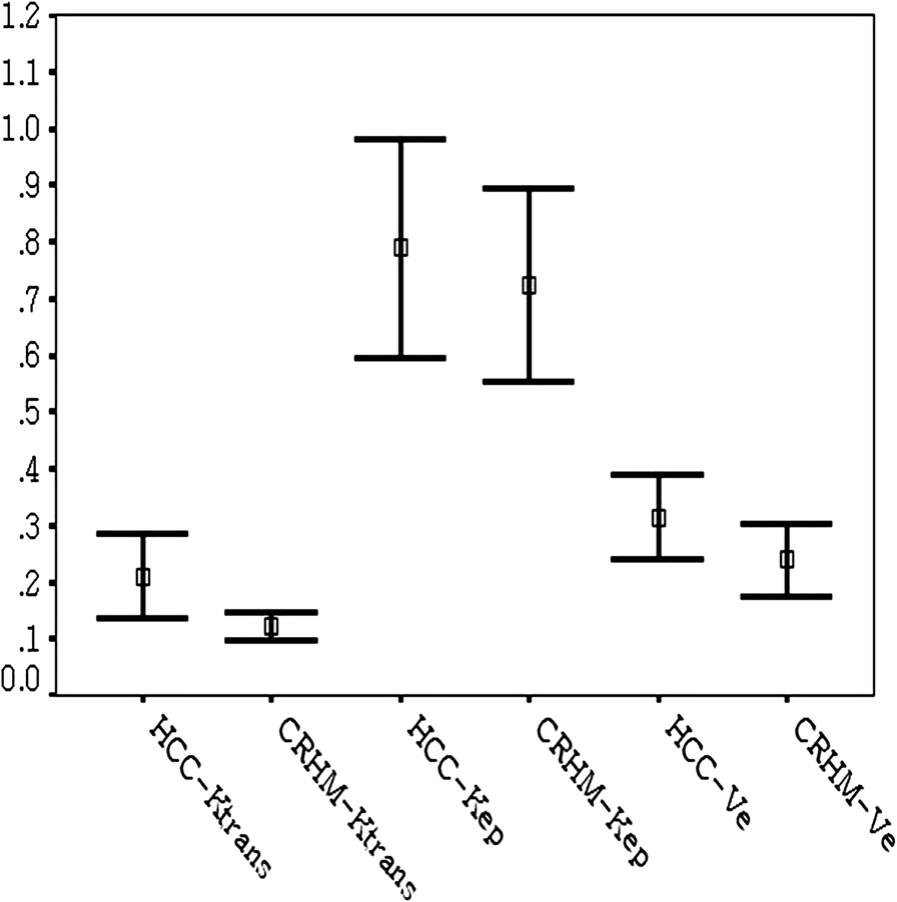
Mean and 95% confidence interval for perfusion parameters (Ktrans,Ve,Kep) for HCCs and CRHMs.

All hepatic tumors were successfully evaluated using the software tool. The mean differences and their analysis using the Bland-Altman plots are summarized in Table 1. Results of the inter-observer agreement analyses are also represented graphically by Bland-Altman plot (Figure 3a-c). Significant differences between HCCs and CRHM were found for the mean K^trans^ as determined by both readers (p = 0.017), but no significant differences were found for V_e_ (p = 0.117) or K_ep_ (p = 0.595). Distribution of the mean perfusion parameters (K^trans^, V_e_, K_ep_) is shown in Figure 4.

**Table 1:** Summary of results of perfusion paremeters obtained for HCCs and CRHMs by Bland-Altman plots

	**Mean difference (SD)**	**95% limits of agreement**	
		**Lower limit**	**Upper limit**
K^trans^	−0.0048 (0.0281)	−0.0598 (−0.0794 to −0.0402)	0.0502 (0.0306 to 0.0698)
V_e_	−0.0031 (0.0378)	−0.0771 (−0.1036 to −0.0507)	0.0710 (0.0446 to 0.0974)
K_ep_	−0.0630 (0.2436)	−0.5405 (−0.7109 to −0.3702)	0.4145 (0.2441 to 0.5849)

## Discussion

Traditional cross-sectional tumor imaging focuses solely on tumor morphology. With the introduction of targeted biological therapies in human trials, morphologic changes as seen on imaging may lag behind other physiologic responses that were previously not measurable. Perfusion MR imaging is a new imaging method that enables characterization of microvascular structures and permeability *in vivo*[10,11] and is emerging as a promising method for monitoring tumor response to treatment [12]. Imaging the entire liver for perfusion analysis of a hepatic tumor appears mandatory, given that most tumors are of heterogeneous morphology, with variable degrees of tumor vascularity, necrosis, or hemorrhage. A single slice image, as used in one previous study [7], is therefore suboptimal as representation of the tumor in its entirety.

## Conclusions

The Bland-Altman analysis indicates that the perfusion parameters (K^trans^, V_e_, and K_ep_), as determined in our study using semi-automatic measurement software, are reproducible and accurate. We conclude that the perfusion parameters evaluated by the software were not significantly influenced by observer experience. The perfusion MR images of all patients in our study reached the adequate quality necessary to obtain measurements. Respiratory motion is a major issue in hepatic perfusion imaging. In our study, all the patients held their breath during the different phases of MR scans, and image acquisition was undertaken by carefully trained and dedicated technicians. After motion correction and registration by the software, the perfusion MR images were free of motion artifacts and misregistration. Good scanning technique, careful patient positioning and reproducible injection technique are important for reproducibility of perfusion MR evaluation. Galbraith [13] et al. evaluated 16 patients with various tumors who underwent MR examination within 1 week of each other and assessed perfusion parameters (K^trans^, K_ep_, and V_e_) using the Tofts’ model, concluding that V_e_ had good reproducibility within individuals, whereas K^trans^ and K_ep_ were more variable, but nevertheless could detect changes in tumors ranging from −14 to +16%, and ±16 or ±17%, respectively. Ng [4] et al. reported that the rate of reproducibility of DCE-MRI parameters was in the range of 10%-20% and is influenced by lesion location, parameters being significantly more reproducible in the liver than in lung tissue.

The transfer constant (K^trans^) is a mathematical function which describes the relationship between the AIF and contrast concentration changes occurring in the voxel [14], which is a parameter related to vessel permeability and tissue blood flow. Our study showed that the mean values of K^trans^ obtained from the HCCs were significantly higher than those obtained from CRHM. This is consistent with findings by Ueda [15] et al., who report that the vascular volume of metastases is generally smaller than that of HCCs. Tumor angiogenesis may serve as independent prognostic indicator and has been shown to predict clinical outcome in patients with lung, breast and colon cancer [16-18]. Vascular endothelial growth factor (VEGF) was discovered as a major driver of tumor angiogenesis. Preclinical studies have shown that anti-VEGF therapy changes tumor vasculature towards a more “mature” or “normal” phenotype. This “vascular normalization” is characterized by attenuation of hyperpermeability, increased vascular pericyte coverage, decrease in basement membrane abnormality, and a resultant reduction in tumor hypoxia and interstitial fluid pressure, which enhances efficiency of drug delivery [19]. The K^trans^ is thought to be uniquely suited to evaluate this normalization process in tumor vasculature [20]. Hence, a decline in K^trans^ after anti-angiogenic treatment reflects either a drop in vascular permeability, vessel density, or both [19]. The clinical trial employing PTK787/ZK 222584 therapy in patients with advanced colorectal cancer and liver metastases reported a rapid reduction in K_i_ (K^trans^) within 26 to 33 hours after the first dose [7]. Similar results have been reported in a trial that involved HCC patients who received sunitinib therapy [21]. Because of the ability to differentiate between HCC and CRHM using the K^trans^ value, we pose that our technique of whole-liver perfusion MR is uniquely suited to be applied during anti-angiogenic treatment. In a tumor environment with permeable vasculature, the V_e_ parameter serves as a marker for tumor necrosis, as well as being inversely correlated to tumor cellularity, which in itself may be an under-utilized indicator of treatment effect [22]. In our study, there was no difference in V_e_ value between HCCs and CRHM, possibly attributable to the high tumoral content and similar volumes of extravascular extracellular space between these tumor types.

The number of patients included into our study was relatively small. In addition, we did not correlate the MR perfusion measurements with histopathological findings, such as microvessel density, and we did not evaluate the response to anti-angiogenic therapy using perfusion MR as an imaging biomarker. However, the primary aim of our study was to assess the feasibility of perfusion imaging using 3D whole-liver MR perfusion in malignant hepatic neoplasms.

Our study suggests that 3D whole-liver MR perfusion enables relevant evaluation of hemodynamic parameters and vessel permeability of hepatic tumors with acceptable inter-observer agreement. These findings demonstrate the ability of this novel technique to contribute to the treatment of hepatic malignancies.

## Abbreviations

3D: Three-dimensional; HCC: Hepatocellular carcinoma; CT: Computed tomography; MR: Magnetic resonance; EES: Extravascular extracellular space; CRHM: Colorectal hepatic metastases; FA: Flip angle; VIBE: Volumetric interpolated breath-hold examination; AIF: Arterial input function; VEGF: Vascular endothelial growth factor; ROI: Region of interest.

## Competing interests

The authors declare that they have no competing interests.

## Authors’ contributions

MSZ, XDQ, SXR designed the research; SXR analyzed the data and wrote the manuscript. CZC and HL were both involved in the acquisition of the data. All authors read and approved the final manuscript.

## Pre-publication history

The pre-publication history for this paper can be accessed here:

http://www.biomedcentral.com/1471-230X/13/53/prepub
